# The Use of Suggestion in the Practice of Dentistry

**Published:** 1918-06

**Authors:** R. E. Denny

**Affiliations:** Philadelphia


### THE USE OF SUGGESTION IN THE PRACTICE OF DENTISTRY

BY DR. R. E. DENNY, PHILADELPHIA

I want to talk to you tonight about Suggestion, the thing that makes diplomats great, the thing that makes salesmen successful, the thing that makes every one who consciously employs it greater and more powerful in his influence with his fellow men.

Suggestion in the broadest sense of the word is the influencing of the mind of one individual by another.

More definitely stated, it is the intrusion into the mind of an idea, met at times with more or less resistance but acted upon at last unreflectively—almost automatically. Hypnotic Suggestion is the accomplishment of this while the subject is in a hypnotic state. With Hypnotic Suggestion we as dentists have little need. There are so many reliable anaesthetics as to make its use for that purpose entirely unnecessary. The popular although erroneous idea that once hypnotized the subject remains more or less under the influence of the hypnotist ever after, would also rise to distrust on the part of the patient and destroy that confidence which is so essential to the successful rendering of those difficult services which we are continually being called upon to give. But we have much use of suggestion when the individual is in a normal state, and a thorough understanding of how to employ it would solve many of the problems which confront us in the management of our patients.

### DIRECT OR INDIRECT

By direct suggestion is meant a clear, definite, statement or command such as "You can not now feel pain," or "You can not now bend your arm." The greater the inability to co-ordinate thoughts and actions, the more effective is direct suggestion and it is therefore more effective in cases of hypnotism and drunkenness.

By indirect suggestion is meant the employment of suggestion by inference. I hold a newspaper in my hand and begin to roll it up; soon I find that my absent-minded friend sitting opposite me is rolling his up also. This is indirect suggestion at work. I hear someone hum a few bars of an old song; shortly after I find myself absent-mindedly whistling the same tune. Coming back to the realization of what I am doing I wonder what started me on that almost forgotten air. These are examples of indirect suggestion and it is the kind of suggestion that we can employ successfully in our every-day work.

### CONCRETE EXAMPLES

A child comes to us for the first time—the mother on the way to the office has attempted in her well meant but bungling way to prepare the child for her visit by such statements as, ''You mustn't cry, he won't hurt you,'' ''He won't pull any of your teeth,'' and like expressions. Is it any wonder that we have a frightened little creature for a patient? The child asks itself, ''Why does mother say—'Don't be afraid,'' and, 'It doesn't hurt?' It must be because it's something terrible the dentist does to little children.'' The mother has unintentionally suggested terrible thoughts to that little mind. Such a mother should certainly be excluded from the operating room or made to keep silent. If on the other hand she is a wise mother and tells her little one, ''We are going to visit Dr. Smith this afternoon, he has the most interesting things to show little folks—a little electric light no larger than the end of your thumb, a chair that lifts you ever so high and lets you down like an elevator. He has little magic balls of mercury that you can't pick up with your fingers, and if you drop them, you can never find them again.'' This mother has here suggested that a dentist's office is a *pleasant* place to go. The first visit will be made with eagerness and not with fear and great reluctance.

### THE WRONG WAY

If the dentist receives the patient indifferently; with untruths and statements such as ''I'm not going to hurt you, I'm not going to pull any teeth;'' and upon signs of tears in the little eyes, ''Don't be a coward now,'' or, if real sobs come when real pain is inflicted, ''You're the biggest baby I ever saw, your mother will just have to hold your hands.'' What terrible depressing suggestions the little child has been given. It has been made to feel that the dentist is a monster and the child itself is a spineless coward. Is it any wonder then that it should act like one?

### THE RIGHT WAY

On the other hand, if the dentist consumes a few minutes of his time in making the child feel at ease, showing the tiny light, the elevator chair, the magic balls of mercury, then asks if he would like to get up into the chair like father does and have his teeth looked at, then after showing in the mirror the stains that mar the beauty of the little white teeth—it is not difficult to create a desire to have clean white teeth. Don't you think the suggestions you have made to that little mind are favorable to you? At subsequent visits, when some difficult and perhaps painful work is to be attempted, the courage of the child should be bolstered up by such suggestions as, "It certainly is a pleasure to have such a manly little fellow as a patient." "He doesn't say a word if it isn't pleasant because he knows that I won't do any more of that than I can possibly help." "I am going to tell father the very first time I see him what a fine little patient you are," and, as you inflict pain, "I know that doesn't feel pleasant, but I am so glad you are a little man and can stand it," etc. Never if possible push the work to a point where their courage will be broken and they will break down in sobs. If this should happen, it is part of wisdom to stop for that day. Puff them up till they nearly burst with pride, and it will stiffen their backbone and give them courage to endure.

### GROWN-UPS, TOO

We must not forget that adults are also subject to suggestion, but it must be much more delicately used. When they say, "I am so nervous, Doctor," you reply, "Of course you are, that is characteristic of highly intelligent people. You can cut a jelly fish into bits and it probably wouldn't suffer at all, but a piece of paper flying across the road will terrify a thoroughbred horse. Highly intelligent people are far more susceptible to nervousness than duller, stupid ones. It is not a mark of cowardice to be nervous, but to resolutely do those things which we have to do, even though our hearts quake within us, is a real mark of courage, and that is what you show every time you have a cavity prepared and stand it as beautifully as you do.'' Don't you think you have given renewed courage to that person? How much inspiration could a patient get from, ''You haven't much nerve, have you?''

### UNDER ANESTHETICS

If, while our patient is in the early anesthetic state we remark to our assistant that the inhaler does not work well, or that the gas is not flowing smoothly, or that the patient does not take the anesthetic well, the suggestion in these remarks is exaggerated in the patient's mind, and he is very prone to become excited, struggle, cry, scream, or even fight in his excitement.

If, on the other hand we say to our patient, ''Please give me your utmost confidence—banish from your mind all thoughts of fear—remember that I have given this anesthetic hundreds of times and that you have every reason to trust me implicitly—the success of the operation depends upon your mind offering no resistance.'' Then as they begin to take it, encourage them with such suggestions as, ''You are taking it splendidly. You couldn't do better if you tried,'' and similar encouraging suggestions until the analgesic stage is reached. Then direct suggestion may be employed. State positively that, ''While you feel me working you can feel no pain,'' ''Take a little nap and leave all the responsibility to me.'' If your patient will do this, drift off and leave the whole job to you, success must follow. Suggestion of this kind means everything in analgesic work. With resistance on the part of the patient indifferent results will follow.

### ITS MONEY VALUE

Indirect suggestion may be employed in many ways in our work. Certainly it may be used effectively in the selling of our services. If a patient asks the price of a denture and we say $25.00 hesitatingly—in a way that would suggest that we feel we are asking more than it's worth, we immediately raise the question in the patient's mind as to whether or not we are charging too much. But if we first call their attention to the wonderful improvement that has been made in plates, in the superior fit that we are able to obtain by improved methods of impression taking, the greater ability to masticate because of the modern artificial teeth that we always use, the naturalness of the teeth in form and color compared to those formerly used, and you have prepared the patient for a fee greater than was charged in former years and perhaps greater than you ask.

### PERSONAL APPEARANCE

It is not by speech alone that one may convey suggestion. A man may fairly radiate suggestions by his appearance and his acts. If he is careless and slovenly in his dress, he suggests that he has a poor opinion of himself and surely he could not inspire confidence in others. If he wears loud and splashy clothes he suggests that he is without refinement, cares more for vain display than for the really essential things in life and is therefore not a man to inspire confidence and trust. A man who is carefully and tastefully dressed suggests refinement and culture and one would instinctively feel that an acquaintance with him would be a thing worth while. A man radiates suggestion by his demeanor. If he has a hang dog expression, a slouchy gait, and in greeting gives you a limp hand to shake and hand back to him, he suggests weakness and incompetence. A man who stands erect when he greets you, looks you in the eye with a pleasant smile, gives you a grip that says, "I'm delighted to see you," that man suggests power and ability. Such a man you would be willing to follow through difficult situations.

YOUR OFFICE

An inanimate thing such as a dental office can fairly speak of its owner. If the furnishings of the waiting room are worn threadbare, the table covered with ancient magazines, the equipment of the operating room long out of date and with a general appearance of untidiness everywhere, the patient knows instinctively that the dentist must also be far behind the times. If, however, the office is furnished with taste, the operating room has modern equipment and a general air of cleanliness and tidiness, it suggests that the dentist is abreast of the times and a long step is made towards acquiring the confidence of the patient.

Suggestion is a force that few recognize. As the sun can make the traveler joyfully remove his coat when the blustering wind would make him button it all the tighter, so the subtle force of suggestion will make men do your bidding where force could never prevail.—*Caulk's Quarterly.*

